# Surface-enabled propulsion and control of colloidal microwheels

**DOI:** 10.1038/ncomms10225

**Published:** 2016-01-04

**Authors:** T. O. Tasci, P. S. Herson, K. B. Neeves, D. W. M. Marr

**Affiliations:** 1 Chemical and Biological Engineering Department, Colorado School of Mines, Golden, Colorado 80401, USA; 2 Department of Anesthesiology, University of Colorado, Denver, Colorado 80045, USA; 3 Department of Pharmacology, University of Colorado, Denver, Colorado 80045, USA; 4 Department of Pediatrics, University of Colorado, Denver, Colorado 80045, USA

## Abstract

Propulsion at the microscale requires unique strategies such as the undulating or rotating filaments that microorganisms have evolved to swim. These features however can be difficult to artificially replicate and control, limiting the ability to actuate and direct engineered microdevices to targeted locations within practical timeframes. An alternative propulsion strategy to swimming is rolling. Here we report that low-strength magnetic fields can reversibly assemble wheel-shaped devices *in situ* from individual colloidal building blocks and also drive, rotate and direct them along surfaces at velocities faster than most other microscale propulsion schemes. By varying spin frequency and angle relative to the surface, we demonstrate that microwheels can be directed rapidly and precisely along user-defined paths. Such *in situ* assembly of readily modified colloidal devices capable of targeted movements provides a practical transport and delivery tool for microscale applications, especially those in complex or tortuous geometries.

## Abstract (web summary)

Most artificial microdevices include biomimetic features, but existing systems do not yet match living microorganisms in speed and direction control. Here, Tasci *et al.* use a rotating magnetic field to reversibly assemble colloidal particles into wheels that translate at velocities approaching 100 μm s^−1^.

## Graphical abstract

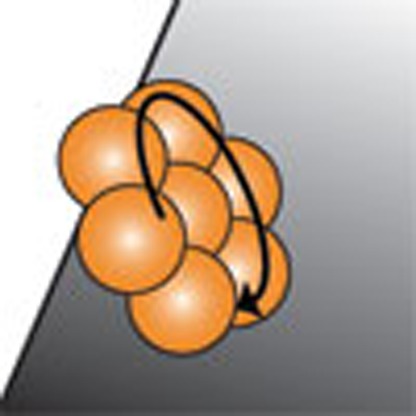

At the microscale, fluid dynamics are unique because viscous forces dominate over inertial forces, a condition typically characterized by Reynolds numbers (Re) less than unity. Because propulsion schemes that rely on inertial forces cannot be used, translation requires approaches adapted to overcome the inherent reversibility of low-Re flows by breaking symmetry^1^,^2^,^3^. For example, microorganisms use undulating or rotating flagella and cilia for motility. In the push for technological devices small enough to move through microscale channels (10–100 μm) over macroscale distances (>1 cm), such as those found in human vasculature, there is appreciable effort in developing equivalent artificial approaches. Propulsion schemes based on catalytic methods^4^,^5^,^6^ or on cellular machinery analogues^7^,^8^,^9^ have shown good progress; however, significant challenges remain. Though catalytic swimmers can reach high speeds^10^, they require available solute fuel for propulsion and concentration gradients for direction^11^. While top–down fabrication of flagella has led to speeds comparable to some microorganisms, they cannot be reconfigured for applications in dynamic or varying environments.

Colloidal assembly is a promising alternative. These methods provide bottom-up fabrication using simple colloidal building blocks as components of microstructures that are rapidly and reversibly assembled into a variety of sizes and shapes. Fabrication is initiated via specific^12^,^13^ or non-specific^14^ interactions or supplemented with applied electrical^15^, optical^16^ or magnetic fields^17^ to enable switching and direct control of size, structure and function^18^. Used with superparamagnetic colloids, magnetic fields are well-suited to assemble structures *in situ* that are easily manipulated and rapidly disassembled after use. Fields of only a few milli-Tesla (mT) create sufficient dipole strength to induce colloidal assembly. Static applied fields align particles into chains, while rotating fields create net isotropic interactions that can lead to compact aggregates^19^. In our studies, we use superparamagnetic colloids and balance magnetic and viscous forces with appropriate field strengths and rotational frequencies to create reversible close-packed assemblies that subsequently spin due to their net dipole interacting with the dynamic applied field^20^,^21^. While rotating magnetic fields can construct microwheels and create a driving torque, the reversible nature of low-Re flows dictates that spinning symmetric objects suspended in fluid do not translate. For net movement to occur, symmetry, either in the device or in the surrounding geometry, must be broken. In an approach particularly appropriate for microenvironments where surface to volume ratios are high and surfaces are plentiful, one way to break the symmetry is with a nearby wall.

Here, we show that rotating magnetic fields can be used to assemble and spin microwheels that, when canted relative to the surface, roll smoothly and quickly with a high degree of directional control. We demonstrate this propulsion mechanism with microwheels composed of 1, 2, 3, 7 and even 19 paramagnetic colloidal particles with translation speeds >100 μm s^−1^. These results demonstrate a rapid and reversible microdevice assembly and powering method that overcomes many of the limitations inherent in biomimetic artificial micropopulsion strategies.

## Results

### Microwheel assembly and translation mechanism

**Figure 1:**
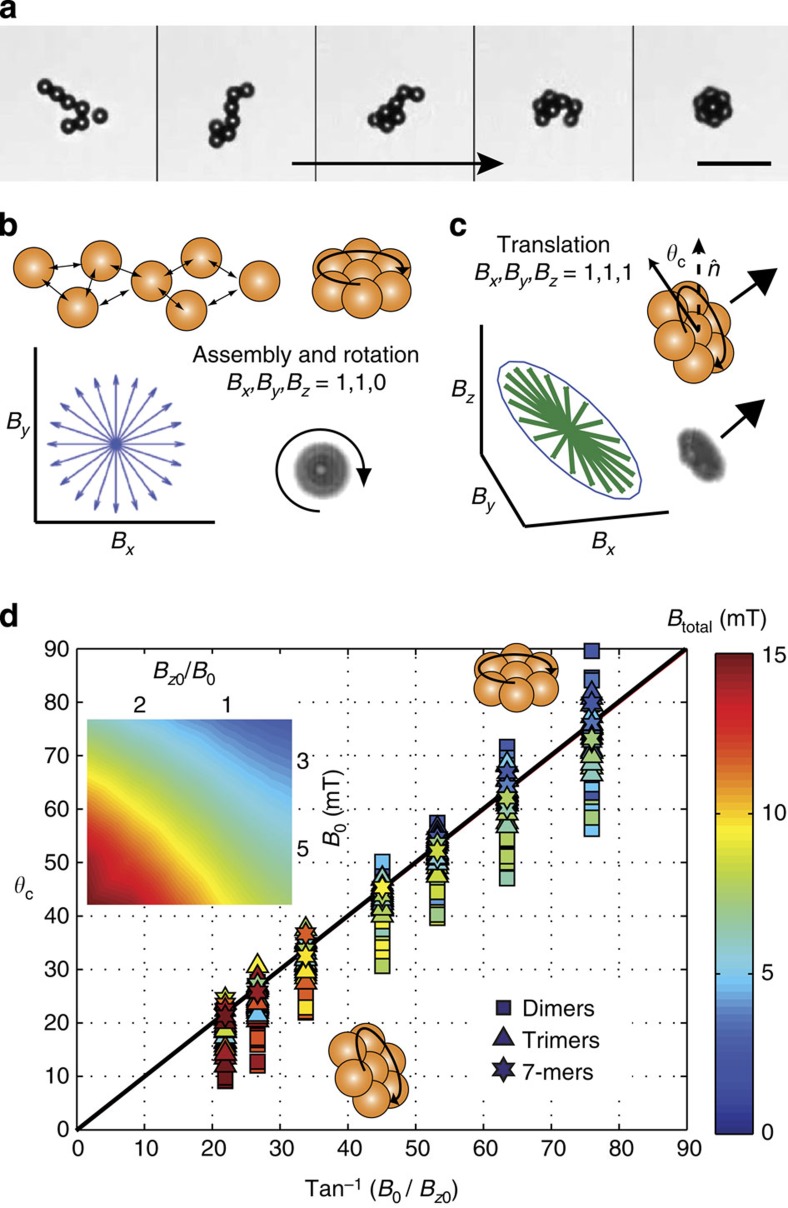
Field-induced assembly and rotation. (**a**,**b**) With application of the rotating magnetic field *B*_*x*_+*B*_*y*_ in the surface plane, colloids assemble via isotropic interactions and ‘sit and spin' (scale bar, 20 μm). (**c**) With addition of a normal variable-phase component (*B*_*z*_), the field rotation axis is oriented towards the surface plane, wheels ‘stand up' at a camber angle, *θ*_c_, and roll along the surface. (**d**) *θ*_c_ measured during wheel translation as a function of the applied field rotation axis set via tan^−1^(*B*_0_/*B*_*z*0_). Data points (*n*=368) are coloured by the magnetic field magnitude 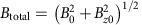 from low (blue) to red (high). The black line corresponds to perfect alignment between microwheel and field rotation axis. The inset identifies total field magnitudes as field ratios are varied.

We begin by setting the in-plane field magnitudes equal as *B*_*x*0_=*B*_*y*0_=*B*_0_ varied sinusoidally to create a circularly rotating magnetic field in the *xy* plane: *B*_*x*_=*B*_0_ cos (*ω*_*f*_*t*−*φ*_*x*_) and *B*_*y*_=*B*_0_ cos (*ω*_*f*_*t*−*φ*_*y*_) where *ω*_*f*_=2*πf* is the field angular frequency, *f* the field frequency, and *φ*_*x*_ and *φ*_*y*_ are phase angles with *φ*_*x*_−*φ*_*y*_=*π*/2. Superparamagnetic beads assemble into microwheels by isotropic interactions induced by the in-plane rotating magnetic field (Fig. 1a,b) with wheel size controlled by local bead density. Spinning microwheels lying flat on a surface have no net motion. For translation to occur they must be inclined relative to the surface; therefore, to propel microwheels, we introduce a normal component to the magnetic field to orient the field rotation axis towards the surface plane (Supplementary Movies 1 and 2). With addition of a field in the *z* direction, *B*_*z*_=*B*_*z*0_ cos (*ω*_*f*_*t*−*φ*_*z*_), both symmetric and asymmetric microwheels reorient off the surface to a defined camber angle, *θ*_c_ (Fig. 1c,d), and begin to translate. Apparent in this approach is the similarity of microwheels to rolling tires where friction with the road, combined with tire rotation, propels wheels forward. One difference between microwheels and tires is that the camber angle can vary from lying flat, *θ*_c_*=*90°, and spinning without translation to fully upright, *θ*_c_*=*0°, and rolling (Fig. 1b,c). Important parameters influencing the rolling velocity *V* include the number of particles comprising the microwheel *n*, its angular frequency ω, and, as wheels rotate, the outer circumferential velocity *V*_*ω*_>*ωR*.

**Figure 2:**
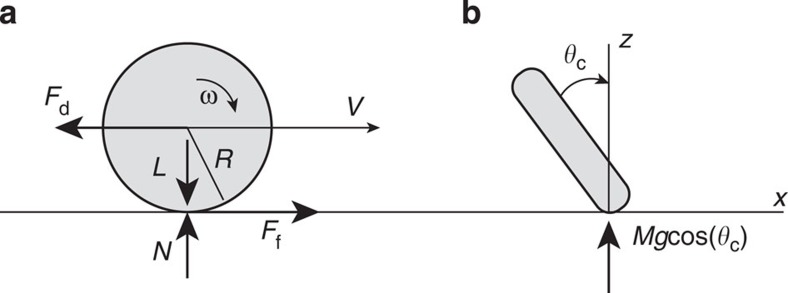
Scaling analysis. (**a**) Side view and (**b**) front view of translating microwheel modelled as a disk. The important parameters include *F*_d_, drag force; *F*_f_, friction force; *L*, load; *N*, normal force from wall; *M*, mass of the wheel; *θ*_c_, camber angle; *ω*, angular frequency; *g*, gravitational constant; and *R*, radius.

For tires, the rolling velocity, *V*, and circumferential velocity, *V*_*ω*_, are almost equal, a fact used in an automobile speedometer to gauge speed and distance travelled. Microwheels however spin much faster than they roll, suggesting a significant fluid layer between the wheel and wall that warrants an approach based on wet friction rather than dry friction. To clarify the mechanism of rolling velocity as a function of angular frequency, we use a force balance in the normal and rotational directions (Fig. 2). First, a sum of forces in the normal, *z* direction:


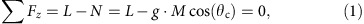


where *F*_*z*_ is the force in the *z* direction, *N* is the normal force, *g* is the gravitational constant, *M* is the mass, and the *L*, is the load. Solving for the load gives


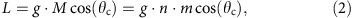


with *n* the number of particles in a wheel and *m* the buoyant mass of a single particle. For the rolling, *x* direction, we have


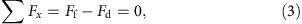


where *F*_f_ is the friction force and *F*_d_ is the drag force. Since circumferential velocity is greater than translation velocity, *V*_*ω*_>*V*, the drag and friction forces oppose one another. We use


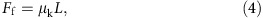


with *μ*_k_ as the wet friction coefficient, which is referred to variously as the fluid friction, viscous friction, or the hydrodynamic lubrication region in the context of load and friction in bearings. Petroff first examined this situation and found that the wet friction coefficient is proportional to velocity after balancing frictional torque with that torque required to shear the intervening fluid layer^22^. Following his approach, we begin by approximating the fluid shear stress as


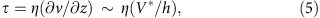


where *η* is the dynamic viscosity of the fluid, *v* is the velocity vector, V* is the wheel edge velocity and *h* is the gap between wheel and surface. This shear stress corresponds to a torque, *T*,


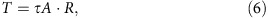


with *A* as the contact area and *R* as the wheel radius. Recognizing that the frictional torque, *T*_f_, is


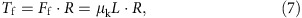


and equating the two torques leads to


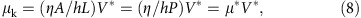


with pressure *P=L/A* and defining μ*≡*η*/(*hP*). With V* the fluid velocity between the wheel and wall=*V*_*ω*_−*V* and approximating 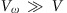, we calculate the friction force as


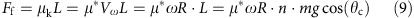


To approximate drag force during translation, we neglect the presence of the wall and employ low Re results for the edge-wise drag on a disk^23^


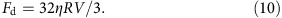


Using this, we equate the drag force and frictional force and solve for the rolling velocity


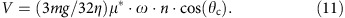


We define a weighted angular frequency, ω*, as


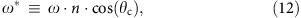


and then can write the rolling velocity as


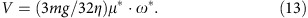


### Microwheel rolling

**Figure 3:**
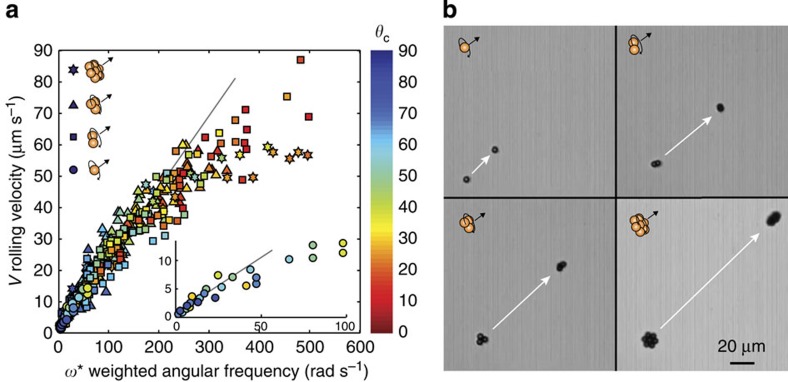
Wheel rolling. (**a**) Rolling velocity for microwheels created from 1 (*n*=25), 2 (*n*=168), 3 (*n*=140) and 7 (*n*=35) colloidal particles as a function of weighted angular frequency, ω*≡*ω*·*n*·cos (*θ*_c_). Data points coloured according to orientation with upright (red) to lying nearly flat (blue). Line indicates the slope (*3 mg/32η*) based on wet friction scaling arguments. To compare results for spherical monomers with those for disk-like wheels, we use the drag force for spheres at low Re, *F*_d_=6*πηRV* resulting in *V*=(*mg*/6*πη*)μ*·*ω*·*n*·cos (*θ*_c_). We therefore scale the monomer results by 32/(3·6*π*)=16/9*π* inset shows unscaled monomer data. (**b**) Three seconds of translation under identical field conditions demonstrate that larger wheels roll faster (Supplementary Movie 3). Supplementary Movies 4–6 show that increased angular frequency and lower camber angles lead to increased speeds as well.

Figure 3a shows the rolling velocity, *V*, as a function of a weighted angular frequency, ω*, that accounts for microwheel size and angular frequency. The scaling argument presented above predicts a slope of (3*mg*/32*η*)μ* on plots of *V* versus ω*. The data show good agreement with this scaling for weighted angular frequencies below 200 rad s^−1^. Above this the scaling deviates from the data, likely because the assumption that the wheel velocity is much greater than the fluid velocity is no longer valid. Wheels roll along the surface at speeds of up to 90 μm s^−1^ with applied field frequencies up to 50 Hz over the range 0<*θ*_c_<90°. Even single particles roll as long as the surface-parallel component of the rotational axis is non-zero. Wheels composed of 2, 3, 7 and even 19 particles, though not strictly round, exhibit smooth motion as they rapidly spin and translate across flat surfaces for values of *B*_*z*0_*/B*_0_<2.5 (Fig. 3b; Supplementary Movies 3–6); at higher values motion becomes unstable. Velocities >120 μm s^−1^ were achieved with dimers and 19-mers at higher amplitude fields, but observation times were limited due to the size of the field-of-view.

As the data condense to a single line at low to moderate *ω* in Fig. 3, we can conclude that friction is not stick-slip and wheel speeds increase with wheel size for a given angular frequency due both to the increased load and increased fluid velocities near the wall. A useful feature of this approach is that different sized microwheels, and thus different speeds for a given field rotation frequency, can be assembled from the same building blocks by changing the bulk colloid concentration. In fact, wheels composed of particle numbers other than those studied in Fig. 3 do roll as well; however, structural isomers in these systems make quantification difficult.

### Directional control of microwheels

**Figure 4:**
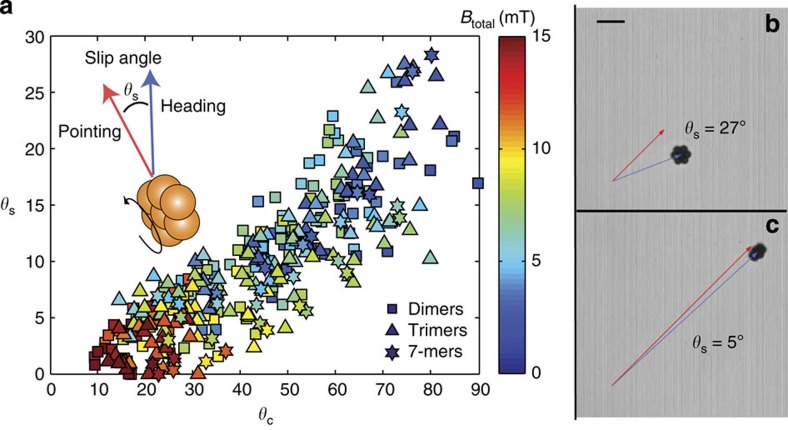
Side slip. (**a**) As *θ*_c_ increases, heading and pointing directions separate as characterized by increasing wheel side slip angle *θ*_s_ (*n*=343). (**b**) *θ*_c_=76°, *V=*14 μm s^−1^ (scale bar, 20 μm), (**c**) *θ*_c_=28°, *V*=39 μm s^−1^.

Targeting applications require not only microwheel propulsion but also the ability to direct them to desired locations. Unlike tires, microwheels can be oriented at very high *θ*_c_ and, as a result, can experience significant lateral forces and side slip. Defining the side slip angle, *θ*_s_, as the difference between the rolling direction (heading) and the wheel rotation plane (pointing) directions we observe that lateral forces push wheels towards the wheel rotational axis (Fig. 4). The influence on side slip on heading is best illustrated by an example; Supplementary Movie 7 shows two wheels programmed to make a circular motion, where one wheel has a camber angle that causes it to lean inward towards the centre of the circle and another has a equal and opposite camber angle that causes it to lean outward. The wheel that leans inward gives a tighter circle compared to the wheel that leans outward.

**Figure 5:**
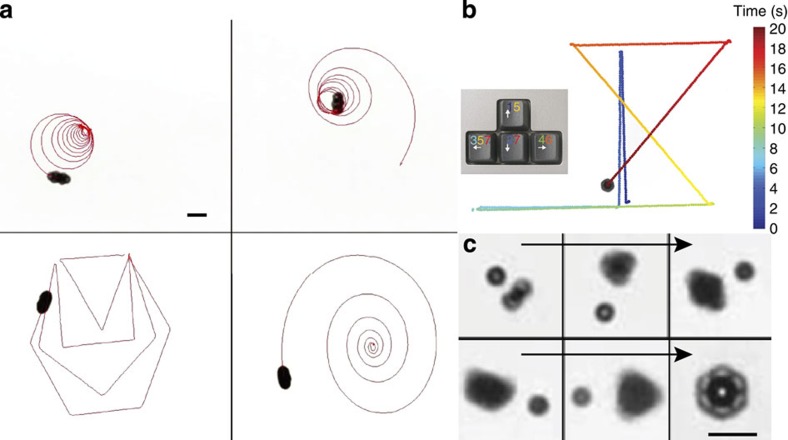
Directional control. Supplementary movies demonstrate (**a**) automated patterns (Supplementary Movie 8; scale bars, 10 μm), (**b**) manual control (Supplementary Movie 9) and (**c**) stepwise microwheel assembly (Supplementary Movie 10).

The microwheel heading angle *θ*_*d*_ (the angle between the heading vector and the positive *x* axis) is related to the phase angle of the fields and the camber angle by *θ*_*d*_=(*φ*_*y*_−*φ*_*x*_)+(*φ*_*z*_−*φ*_*x*_)+*θ*_s_=*φ*_*z*_−*φ*_*x*_+*θ*_s_−*π*/2. We can use the heading to redirect microwheels with a simple phase shift in the *z*-field. As a result, speed and heading changes can be actuated immediately in preprogrammed patterns (Fig. 5a; Supplementary Movie 8). Alternatively, the direction of microwheels can be manually manipulated by keyboard controls (Fig. 5b; Supplementary Movie 9). This manual control was used here to assemble microwheels of different sizes, for example the 7-mer in Fig. 5c (Supplementary Movie 10). Note that, during assembly of the 7-mer, asymmetric wheels also translate.

## Discussion

With velocity and directional control, microwheels have potential application for cargo transport in complex or tortuous networks of channels, as in the vasculature and within microfluidic devices where surfaces are ubiquitous. Reversible assembly of microwheels *in situ* has significant advantage over pre-fabricated devices as individual particles are injectable and small enough to pass through blood capillaries or microfluidic channels. Once the applied magnetic field is turned off, wheels disassemble into micron-sized components removable via the body's natural defence mechanisms or filtered out in microfluidic platforms. Here and with a bulk field for assembly and powering, microwheels can be fabricated with mass parallelization and function on walls of high curvature because of their ability to roll at high camber angles. The enhanced mobility is particularly useful in medicine where similar superparamagnetic colloids are used for enhanced imaging or modified with surface or embedded moieties for applications such as drug delivery^24^,^25^ or targeted hyperthermia^26^. Magnetic bead-based methods are also used in microfluidic platforms for immunoassays, DNA hybridization, surface patterning and magnetic mixing, sorting and separations^27^. The magnitude of the magnetic fields we employ is low (typically 2–15 mT), making the approach feasible for use *in vivo* or within distributed microdevices significant distances away from field magnets. Equivalent translation rates by magnetophoresis alone would require extremely high field gradients^28^ and field magnitudes hundreds of times higher than required for rolling.

We note that other approaches use varying magnetic fields to induce translation of small clusters along surfaces^29^,^30^,^31^. For example, asymmetric colloidal dimers have been shown to translate when subjected to a rotating field; however, because of the nature of the field which requires both a.c. and d.c. components and need for cluster asymmetry, velocities are relatively slow (∼10 μm s^−1^). In these experiments, the underlying translation mechanism relies on asymmetric dissipation with the wall resulting in periodic but irregular motion. Importantly, symmetric systems such as monomers do not translate. Here, we exploit a translation mechanism that relies on wet friction using *in situ*-assembled symmetric and asymmetric colloidal wheels to provide smooth motion along surfaces with user-defined directionality and instantaneous reorientation. These features are a direct result of the unique wheel-like structures that provide not only high speeds and directional control but can be immediately dis- and re-assembled unlike non-reconfigurable microstructures propelled by magnetic fields^29^,^30^.

We show that angular frequency, camber angle, and wheel size all drive wheel motion with upper limits to rolling velocity bounded by *ωR*, parameters whose values can be increased with larger field magnitudes. While we present data of velocities approaching 100 μm s^−1^, higher fields, which can be maintained only briefly in our current experimental setup, do lead to velocities >100 μm s^−1^, faster than microfabricated flagella and related techniques^8^,^32^,^33^. While also higher than most microorganisms including *Escherichia coli* at 20–45 μm s^−1^ (refs 34, 35), a number of bacteria have top speeds an order-of-magnitude greater^35^ showing both the effectiveness with which natural flagella can function and how far microfabricated approaches currently lag behind Nature. While there clearly remains work to challenge the quickest microorganisms, our wheel-based rolling may prove a faster, simpler and better controlled microdevice propulsion strategy than swimming in microscale environments where abundant surfaces are available.

## Methods

### Systems studied

We use superparamagnetic Dynabeads M-450 Epoxy (Life Technologies Corporation, Carlsbad, CA, USA) of diameter 4.5 μm, density=1.6 g cm^−3^ suspended in 1% sodium dodecyl sulfate (Sigma-Aldrich) solution. To create a sample chamber, double-sided tape with an 8 mm diameter hole was placed on a glass slide and, after sample was added, sealed with a coverslip. The chamber was then positioned at the centre of the magnetic field system and wheel motion recorded with a microscope (Zeiss Axioplan 2 Imaging) and high-speed camera (Epix SV 643M) operated at 400 frames per s.

### Magnetic field generation

**Figure 6:**
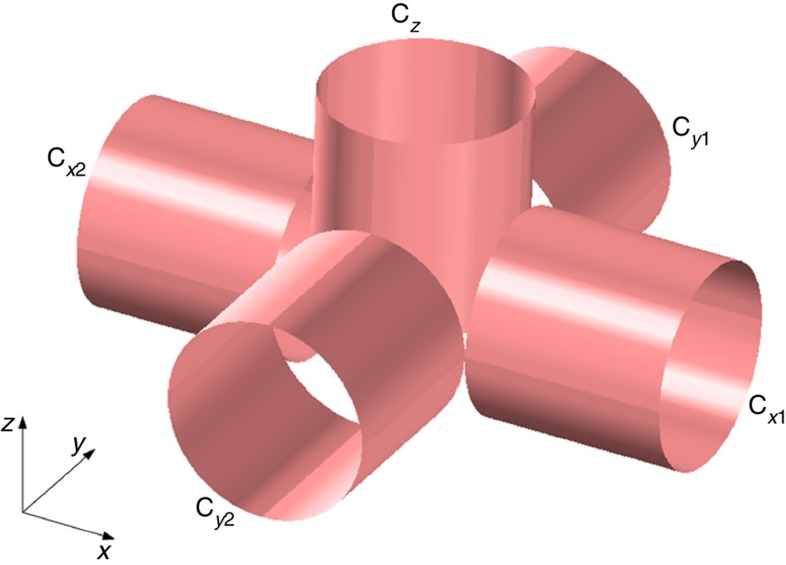
Experimental setup. Magnetic field system consisting of five air-cored solenoid coils.

External magnetic fields were generated with five identical air-cored copper solenoid coils of 50 mm inner diameter, 51 mm length, 400 turns and current capacity 3.5 A (Fig. 6). The field generated at the centre of the experimental setup has three components *B*_*x*_=*B*_*x*0_ cos (*ω*_*f*_*t*); *B*_*y*_=*B*_*y*0_ cos (*ω*_*f*_*t*−*π*/2) and *B*_*z*_=*B*_*z*0_ cos (*ω*_*f*_*t*−*φ*_*z*_). *B*_*x*_ was generated by coils C_*x*1_ and C_*x*2_, *B*_*y*_ was created by C_*y*1_ and C_*y*2_; *B*_*z*_ was generated by coil C_*z*_.

Sinusoidal voltage waveforms were generated using Matlab (Mathworks, Inc., Natick, MA, USA) and an analog-output card (National Instruments, NI-9263) and then amplified (Behringer EP2000) before being applied to individual solenoids. To monitor coil currents, an analog input data acquisition card was used (National Instruments, NI-USB-6009). The resulting magnetic fields were estimated using a custom Matlab code solving the fields of the solenoids for a given current. Predictions of the code were validated by exciting the coils with constant currents and measuring the field with a gaussmeter (VGM Gaussmeter, Alphalab Inc.).

### Data reduction and analysis

**Figure 7:**
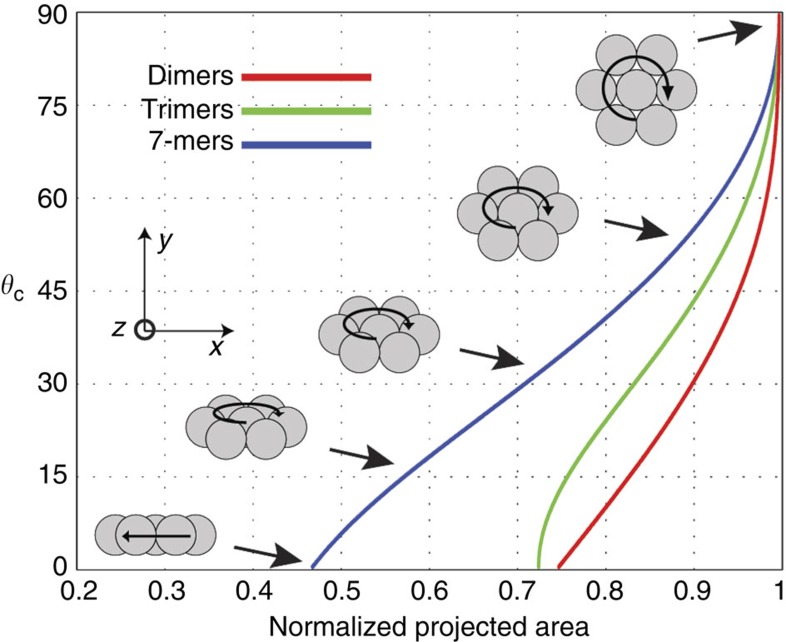
Camber angle standard curves. Camber angle as a function of wheel projected area (viewed from above).

A total of 368 experiments were performed on microwheels composed of monomers, dimers, trimers and 7-mers. In the experiments, different conditions were tested by varying the magnetic flux densities from 2.1 to 15 mT and field frequencies from 10 to 80 Hz. Image processing code was written in Matlab to detect the microwheel assemblies and provide rolling velocity *V*, rotation frequency *f* and camber angle *θ*_c_ (Supplementary Movie 11). Rolling velocity was determined by dividing travelled distance by travel duration. Rotation frequencies were determined by recording pixel sum fluctuations caused by microwheel rotation and taking the Fourier transform. Standard camber angle curves (Fig. 7) were analytically calculated for each microwheel assembly size (dimers, trimers and 7-mers) and used to determine *θ*_c_ for each experiment by measuring the mean projected area occupied by the translating microwheel.

Directional control of the microwheels was performed with custom Matlab code to vary the sinusoidal voltage applied to the *C*_*z*_ coil, create a phase shift of the *B*_*z*_ field *B*_*z*_=*B*_*z*0_ cos (*ω*_*f*_*t*−*φ*_*z*_), and a net field rotation axis reorientation. This shift could be manually controlled with keyboard arrow keys, allowing one to easily direct microwheels to desired locations. With the same code, the path of the microwheels was programmed as shown in Fig. 5a.

### Reynolds numbers

For our systems either a wheel or particle Re can be employed to demonstrate the dominance of viscous over inertial forces. Here, Re=*ρVD*/*μ*, where *ρ* and *μ* are fluid density and viscosity, *V* and *D* are the rolling velocity and diameter of the wheel. One can also consider a particle Reynolds number (Re_p_) where we set *V* to the circumferential velocity *V*_*ω*_ and *D* to the single particle diameter instead. Typical conditions for the experiments presented here have Re∼10^−2^ and Re_p_∼10^−1^; however, at the fastest speeds and largest wheels, Re can approach 0.20.

## Additional information

**How to cite this article:** Tasci, T. O. *et al.* Surface-enabled propulsion and control of colloidal microwheels. *Nat. Commun.* 7:10225 doi: 10.1038/ncomms10225 (2016).

## Supplementary Material

Supplementary Movie 1 Assembly: Spontaneous assembly of wheels upon application of the magnetic field.

Supplementary Movie 2 Rotating Magnetic Field Orientation: Variation of the phase and amplitude of the field *B_z_* can be used to reorient the field rotational axis and camber angle *θ_c_*. With *B_z_* = 0 (left), the field rotation axis is fixed normal to the surface and wheels spin but do not translate. With addition of non-zero *B_z_* (right), wheels spin around the altered rotational axis and translate. Note that all fields are applied at the same frequency and that field simulations and corresponding movies are slowed down for clarity.

Supplementary Movie 3 Size and Rolling Velocity: Translation of wheels from one, two, three, seven, and 19 colloidal building blocks. Larger wheels translate faster for equivalent external field conditions. All videos in real time with apparent spin rate variation due to frame rate and temporal aliasing.

Supplementary Movie 4 Rotation and Rolling Velocity: Dimers held at constant *θ_c_* but increasing *ω* where faster translation is observed. All videos in real time with apparent spin rate variation due to frame rate and temporal aliasing.

Supplementary Movie 5 Camber and Rolling Velocity: Dimers held at constant *ω* but decreasing *θ_c_* where faster translation is observed. All videos in real time with apparent spin rate variation due to frame rate and temporal aliasing.

Supplementary Movie 6 Camber, Rotation and Rolling Velocity: Variation of both *ω* and *θ_c_* for 7-mers. All videos in real time with apparent spin rate variation due to frame rate and temporal aliasing.

Supplementary Movie 7 Camber and Directional Control: Microwheels can be rapidly and precisely directed. Camber angle *θ_c_* influence on heading is apparent in circular patterns where *θ_c_left_* = *−θ_c_right_*. The microwheel on the left leans towards the center of the circular path generating narrower circles while the microwheel on the right leans away from the center to create wider circles.

Supplementary Movie 8 Preprogrammed Control: Various programmed patterns with smooth and sharp turns.

Supplementary Movie 9 Manual Control: Manual control of translation using a keyboard.

Supplementary Movie 10 Stepwise Assembly: Wheel assembly can be directed via keyboard control.

Supplementary Movie 11 Image Analysis: Image analysis was used to measure microwheel rolling velocity, camber angle and rotation frequency. The left window shows the original experimental video and the right the image-processed version. To calculate rolling velocity, the microwheel center (red dot) was recorded. To determine the camber angle, the white pixel count inside the big (embracing) square during translation was used. Rotation frequency was determined from pixel sum fluctuations inside the small square (on the bottom right quarter of the microwheel).
